# Solid Dispersion Matrix Tablet Comprising Indomethacin-PEG-HPMC Fabricated with Fusion and Mold Technique

**DOI:** 10.4103/0250-474X.57290

**Published:** 2009

**Authors:** A. Mesnukul, K. Yodkhum, T. Phaechamud

**Affiliations:** Department of Pharmaceutical Technology, Faculty of Pharmacy, Silpakorn University, Nakhon Pathom 73000, Thailand

**Keywords:** Characterization, drug, release, mold tablet, hydroxypropylmethylcellulose, polyethylene glycol

## Abstract

The purpose of this study is to fabricate the polyethylene glycol matrix tablet by mold technique. Indomethacin and hydroxypropylmethylcellulose were used as model drug and polymer, respectively, in PEG matrix system. The physical and drug release characteristics of developed matrix tablet were studied. This inert carrier system comprising 7:3 polyethylene glycol 4000: polyethylene glycol 400 could effectively enhance the solubility of indomethacin and an addition of hydroxypropylmethylcellulose could sustain the drug release. Scanning electron microscope photomicrograph indicated the drug diffusion outward through the porous network of this developed matrix tablet into the dissolution fluid. Least square fitting the experimental dissolution data to the mathematical expressions (power law, first-order, Higuchi's and zero-order) indicated the drug release kinetics primarily as Fickian diffusion. Both the enhancement of drug dissolution and the prolongation of the drug release could be achieved for aqueous insoluble drug such as, indomethacin, by using polyethylene glycol-hydroxypropylmethylcellulose matrix system prepared with melting and mold technique.

Typically, a tablet dosage form is prepared by compression method. Some special tablet such as orodispersible tablet could be prepared by freeze drying. Melt extrusion has been recently reported as the procedure to produce the drug matrix system for enhancing or retarding the drug release. The main excipient employed for this system should be molten at elevated temperature and could set up as solid matter at room temperature.

Polyethylene glycols (PEGs) are widely used in a variety of pharmaceutical formulations including parenteral, topical, ophthalmic, oral and rectal preparations[1]. Polyethylene glycols are stable hydrophilic substances that are essentially nonirritant to the skin. Polyethylene glycols can also be used to enhance the aqueous solubility characteristics of poorly water-soluble compounds[2]. Liquid grades of PEG 200-600 occur as clear, colorless or slightly yellow-colored, viscous liquids. Grades of PEG 6000 and above are available as free-flowing milled powders that can used as excipient for tabletting. Particularly, PEG 4000 or 6000 has been employed as a carrier for increasing the dissolution rate of several poorly water soluble drugs, such as itraconazole[3], diclofenac[4], prednisolone[5] and rofecoxib[6]. All grade of polyethylene glycol are soluble in water and miscible in all proportions with other polyethylene glycol. Polyethylene glycols do not support microbial growth and they do not become rancid[7]. PEG has potentially utilized as matrix component prepared with fusion and mold method.

Indomethacin is non-steroidal antiinflammatory agent with antipyretic and analgesic properties. It is a nonselective inhibitor of cyclooxygenase (COX) 1 and 2, enzymes that participate in prostaglandin synthesis from arachidonic acid. It has been used in the symptomatic management of painful and inflammatory conditions. It is used in musculoskeletal and joint disorders including ankylosing spondylitis, osteoarthritis, rheumatoid arthritis and acute gouty arthritis. Usual initial dose by mouth in musculoskeletal and joint disorder is 25 mg two or three times daily with food. To alleviate night pain and morning stiffness, 100 mg may be administered by mouth, or rectally as a suppository. In acute gouty arthritis a suggested dose is 50 mg three times daily and in dysmenorrheal up to 75 mg daily has been suggested. Indomethacin is practically insoluble in water, soluble 1 in 50 of ethanol, 1 in 30 of chloroform, and 1 in 40 to 45 of ether, and soluble in acetone[8]. Because of its low water solubility, therefore indomethacin is used as a model drug in the study.

Hydrophilic matrix has become extremely popular in controlling the release rate of drugs from solid dosage forms. When a matrix containing a swellable glassy polymer comes into contact with a solvent, a progressive alteration from the glassy to the rubbery state leads to a swelling process. For matrix system, drug is often released by diffusion, because a sort of receding drug boundary comes to exist within the system[9]. Hydroxypropylmethylcellulose (HPMC) is non-ionic aqueous-soluble cellulose ether derivative for use in controlled-release dosage forms. Owing to high swellability and high gelling strength formation this polymer effectively prolongs drug release, which has a significant effect on the release kinetics of an incorporated drug.

It is interesting to create the system comprising both the drug dissolution enhancement together with the sustained drug release of that dissolved drug in form of matrix system. Therefore, in this study, indomethacin solid dispersion tablets were prepared with melting and mold method using 7:3 PEG4000:PEG 400 as inert carrier and HPMC as hydrophilic polymer to obtain the model formulation having functions of both the drug solubility enhancement and the extended release properties.

## MATERIALS AND METHODS

HPMC type K 15 M, lot No. PH26012N31, (Dow Chemical, Michigan, USA.) and indomethacin (Batch No. 050814, China National Chemical Imp. Exp., Guangzhou, China) were used as received. Polyethylene glycol 4000 (lot no. 504907) and polyethylene glycol 400 (lot no. PO76049) were purchased from P. C. Drug Center Co., Ltd., Bangkok, Thailand. Hydrochloric acid (lot no. E23W60, J. T. Baker, NJ, USA) was used as received. Potassium dihydrogen orthophosphate (lot no. E23W60), di-sodium hydrogen orthophosphate (lot no. 405300), sodium chloride (lot no. AF 407256), sodium hydroxide (lot no. AF 310204) were purchased from Ajax Finechem, Canning Vale, Australia.

### Preparation of tablet by mold technique:

Tablets containing 75 mg indomethacin and different amount of HPMC using PEG4000:PEG400 (7:3) as carrier were prepared with the melting and mold technique. The tablet was prepared by melting PEG 4000 on the water bath and mixed with PEG 400, drug and polymer, respectively, and then the mixtures were poured into the stainless steel mold with diameter of 12 mm.

### Evaluation of physical properties of prepared tablet:

The hardness of the tablets was determined using a hardness tester (Pharmatest, Ontario, Canada). The tablet thickness and diameter were measured using a thickness tester (Teclock, Kyoto, Japan). A test of drug release was undertaken in 900 ml phosphate buffer pH 6.2 using a dissolution apparatus (Erweka DT 70, Heusenstamm, Germany) with the basket method at 100 rpm. Samples were collected at specific time intervals and assayed by a UV/Vis spectrophotometer (Perkin-Elmer, Rodgau-Juegesheim, Germany) at a wavelength of 323 nm. During the drug release studies, the tablets were observed for physical integrity. Drug release from these tablets was compared to capsule containing 75 mg indomethacin powder.

### Dissolution profile fitting:

Least square fitting the experimental dissolution data (cumulative drug release>5% and up to 80%) to the mathematical equations (power law, first order, Higuchi's and zero order) was carried out using a nonlinear computer programme, Scientist for Windows, version 2.1 (MicroMath Scientific Software, Salt Lake City, UT, USA). The coefficient of determination (r^2^) was used to indicate the degree of curve fitting. Goodness-of-fit was also evaluated using the model selection criterion (MSC)[10], given as: $msc=h\left\{\frac{\sum_{i=1}^{n}w_{i}{\left(Y_{obs{\;}_{i}}-{\bar{Y}}_{obs}\right)}^{2}}{\sum_{i=1}^{n}w_{i}{\left(Y_{obs{\;}_{i}}-Y_{cal_{i}}\right)}^{2}}\right\}-\frac{2p}{n},$

where Y_obsi_ and Y_cali_ are observed and calculated values of the i-th point, respectively, and w_i_ is the weight that applies to the i-th point, n is number of points and p is number of parameters.

### Texture analysis:

The swelling behavior of the tablets was investigated through textural analysis. Tablets were placed in the dissolution vessels under condition identical to that described above for dissolution testing. The hydrated tablets were removed at predetermined intervals, and subjected to textural profiling to determine a total work of probe penetration into the entire matrix. All measurements were carried out in triplicate for each time point and tablets were discarded. Textural analysis was performed using a TA.XT2i texture analyzer (Charpa Techcenter, Stable micro Systems Ltd., Godalming, UK) equipped with a 5 kg load cell and Texture Expert software. The force displacement-time profiles associated with the penetration of a 3 mm round-tipped steel probe into the swollen matrices were monitored at a data acquisition rate of 200 points per second. Probe approached the sample at pretest speed of 1.0 mm/s. Once a trigger force of 0.005N was detected (at contact of the probe with tablet) the probe was advanced into the sample at a test speed of 0.5 mm/s until the maximum force of 40 N was reached as described by Baumgartner *et al*[11].

### Differential scanning calorimetry (DSC):

The DSC thermograms of drug, PEG 4000, PEG 400, HPMC, tablet containing 7:3 PEG 4000:PEG 400 without or containing 25% HPMC were obtained using differential scanning calorimetry (DSC) (Pyris Sapphrie DSC, Standard 115V, Perkin Elmer instruments, Yokohawa, Japan). The experiment was done in non-hermetically sealed aluminium pans; the heating rate was 10 °/min and using nitrogen as purge gas (20 ml/min). Samples of approximately 5 mg were weighed into aluminium pans. The heating range was 25-300° and -30-70° with reverse run.

### Determination of surface topography of tablets:

The surface topography of the prepared matrix tablet was determined using scanning electron microscope (SEM) (Maxim 200 Camscan, Cambridge, England). Samples after dissolution test were freeze dried for 24 h to dry the samples and to avoid the collapse of porous structures. The samples were stuck on a metal stub using carbon double adhesive and sputter-coated with gold before test. Micrographs were taken with a scanning electron microscope operated at an accelerating voltage of 20 KeV.

## RESULTS AND DISCUSSION

System comprising 70:30 PEG4000:PEG400 was chosen as carrier for tablet since the tablet prepared by mold technique with this system could be easily remove from the mold and had suitable hardness for tablet dosage form. Hardness of tablet containing different amount of HPMC was increased as the amount of polymers was increased (Table 1). HPMC solution is useful in many industrial applications as a binder, thickener, and to stabilize suspensions and emulsions. It has been reported by many authors that HPMC can be used as an effective excipient for sustained-release systems[12,13].

**TABLE 1:** PHYSICAL PROPERTIES OF INDOMETHACIN TABLETS

Amount of HPMC (%)	Physical Properties			
	
	Weight±SD (g)	Thickness±SD (mm)	Diameter±SD (mm)	Hardness±SD (Newton; N)
0	0.8912±0.0146	6.83±0.21	11.89±0.03	12.84±1.55
5	0.8699±0.0089	6.92±0.31	12.01±0.02	14.59±1.67
10	0.8775±0.0098	6.83±0.21	12.02±0.03	15.09±1.71
15	0.8709±0.0098	6.85±0.20	11.97±0.02	15.74±1.80
20	0.8700±0.0228	6.72±0.12	12.02±0.02	16.44±1.68
25	0.8522±0.0151	6.73±0.19	12.01±0.02	17.89±1.79

Physical properties of 75 mg indomethacin tablet containing 5% w/w xanthan gum and different amount of HPMC in 70:30 PEG 4000:PEG 400 system

**Fig. 1:**
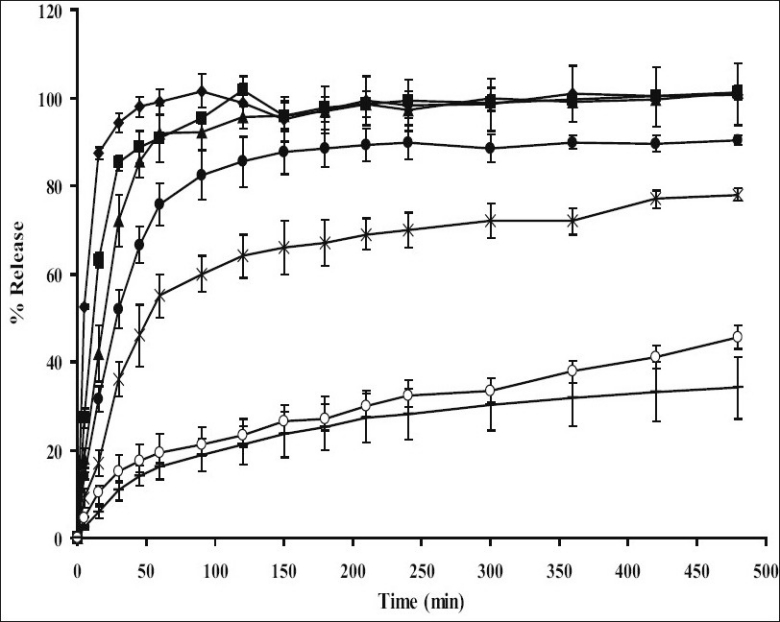
Drug release profiles of indomethacin formulations Drug release profiles of indomethacin from tablets containing (–◆–) 0% HPMC; (–■–) 5% HPMC; (–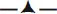
–) 10% HPMC; (–●–) 15% HPMC; (–×–) 20% HPMC; (–○–) 25% HPMC and (–––) capsules in phosphate buffer pH 6.2 (n=3).

The dissolution of indomethacin from tablets containing different amount HPMC is shown in fig. 1. Drug release from tablet without an addition of HPMC was faster than that containing HPMC and capsule because the former tablet contained PEG as a carrier for increasing the solubility of indomethacin. PEGs are one of the most widely used carriers to prepare solid dispersions due to their low melting point and their ability to provide the hydrophilic environment to enhance drug solubility[1]. An enhancement of dissolution rate of hydrocortisone and prednisolone has been reported by means of the fusion method, using sorbitol, sucrose, or PEG 6000 as carrier. The carriers are melted at elevated temperature and the drugs are dissolved in molten carriers[14,15]. To confirm this result, drug release form tablet prepared by solid dispersion method was faster than from capsule containing only indomethacin.

Tablet containing 5% HPMC was faster than tablets containing 10%, 15%, 20%, 25% HPMC and capsule, respectively. High swelling capacity and gel formation of HPMC could retard the release of dissolved drug from the matrix. Relationship between drug release rate and polymer concentration was previously reported by Fu *et al*[16]. As also described by Ghimire *et al*[17], water insoluble drug released from matrix tablets and erosion properties of matrix tablet was found to be dependent upon the HPMC concentration. Such a burst release effect with HPMC for matrices was evident in case of the tablet containing low amount HPMC as also reported by other investigations[18]. It was important to note that this initial rapid release of the drug from the hydrophilic matrix system was often therapeutically undesirable. More amount of HPMC was needed to get a sustained release profile of acetaminophen in the same medium[19]. Drug release from capsule containing only powder of 75 mg indomethacin showed drug release slower than all tablets containing HPMC because indomethacin powder in capsule is a poorly water-soluble drug (5 μg/ml)[3].

**Fig. 2:**
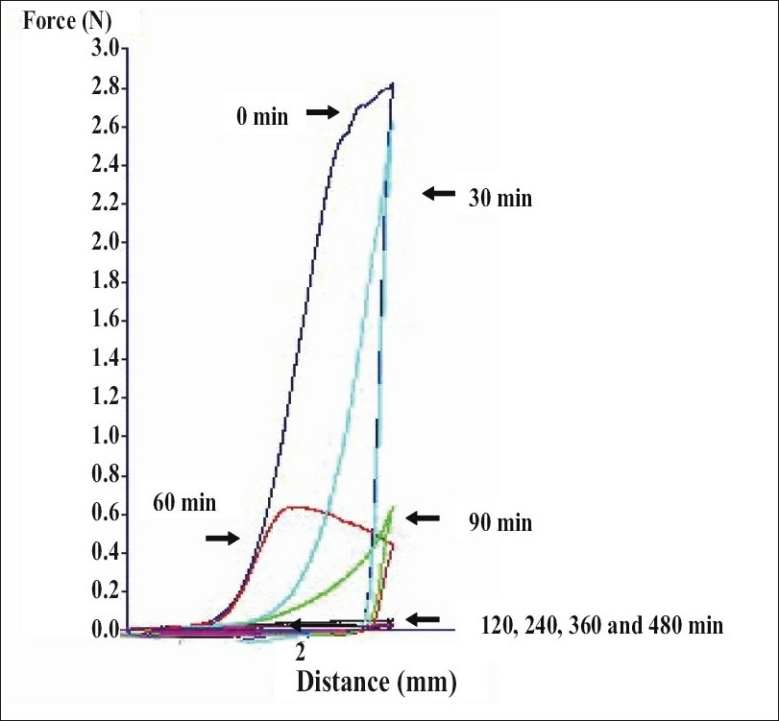
Force displacement profiles of tablets. Force displacement profiles for formulation of tablets containing 25% HPMC in 70:30 PEG 4000:PEG 400 system at different time points (n=3)

**Fig. 3:**
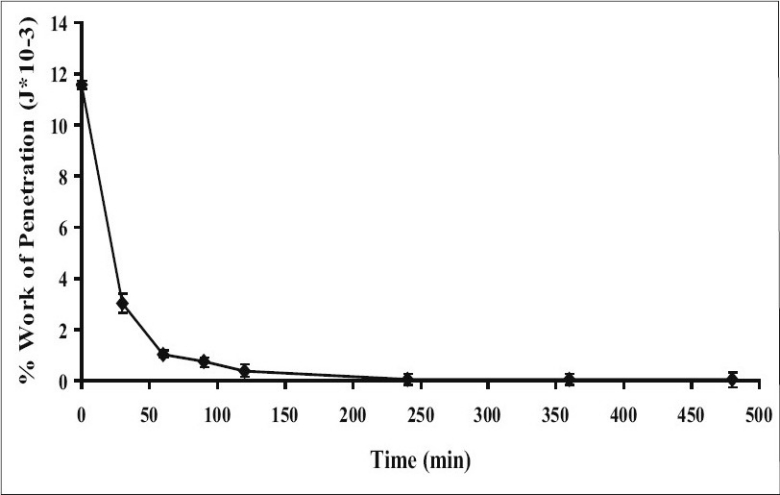
Total work of penetration of tablet with time. Total work of penetration of tablet containing 25% HPMC in 70:30 PEG 4000:PEG 400 system at different time points (n=3)

Dry tablet (before dissolution test) containing 25% HPMC exhibited higher total work penetration than tablet after dissolution test at different time intervals (figs. 2 and 3). Total work penetration of tablet containing 25% HPMC was decreased with time during dissolution test. The dynamic structural changes of the gel layer formed during swelling of tablets containing HPMC were followed by force-displacement measurements (fig. 2). The force required for probe to penetrate the swollen tablet decreased with time as the swelling proceeded and gel strength was reduced. Similar resulted has been reported for the relation between the physical properties of a xanthan matrix in the absence or presence of calcium ions and its influence on the release of pentoxifylline[11]. Force transition regions in a force displacement textural profile of a swollen tablet has been defined earlier[20,21]. The total work of penetration calculated as the area under the force displacement curve indicating matrix stiffness or rigidity[22]. Change in work of penetration versus time of tablets after exposure to medium was extended as the hydration increased. A sharp decrease in work of penetration from 0 h (dry tablet) to 1 h could be observed which reflected the initial high rate of hydration of tablets which incidentally coincided with high rate of water uptake and gel formation. Hydrated tablets demonstrated lower values for work of penetration during 2 to 8 h. Total work penetration of tablet after dissolution test was decreased with time. The total tablet thickness and swelling front movement using force-displacement profiles provided more evidence that the rate and extent of gel formation was significantly influenced by the nature of excipient used[11]. In fact, the obtained results confirmed that the system containing a gel with a lower strength was greater susceptible to erosion and chains disentanglement as mentioned previously[9]. Linear correlations were observed among drug dissolution, polymer content and parameters of texture analysis including hydrogel thickness for formulations containing hydrophilic polyethylene oxide (PEO)[23]. In addition, the results of textural analysis indicated that the total work of penetration was higher for matrix with polymer blend compared to HPMC-only matrix in water and phosphate buffer pH 6.8[24].

**Fig. 4:**
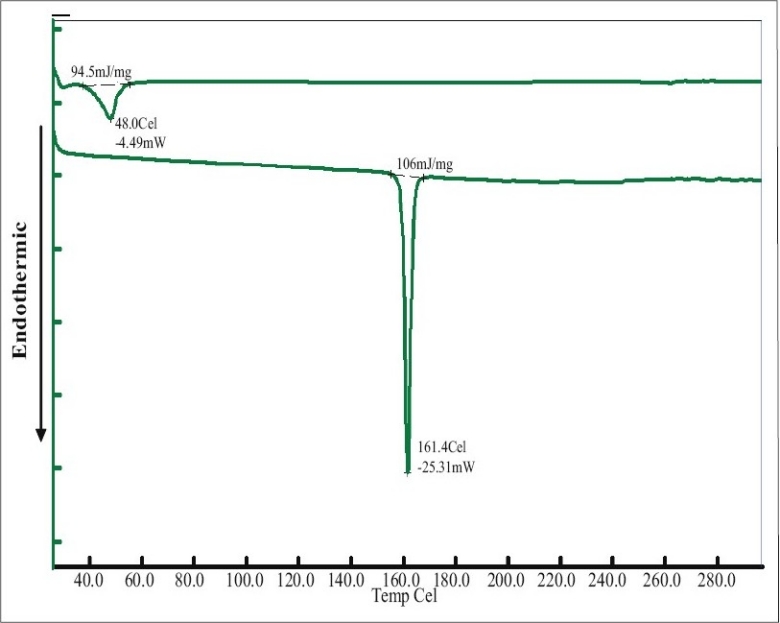
DSC thermograms of PEG 4000 and PEG 400 mixture and indomethacin. Differential scanning calorimetric thermograms of the system containing 7:3 PEG 4000:PEG 400 on the top and indomethacin on the bottom

**Fig. 5:**
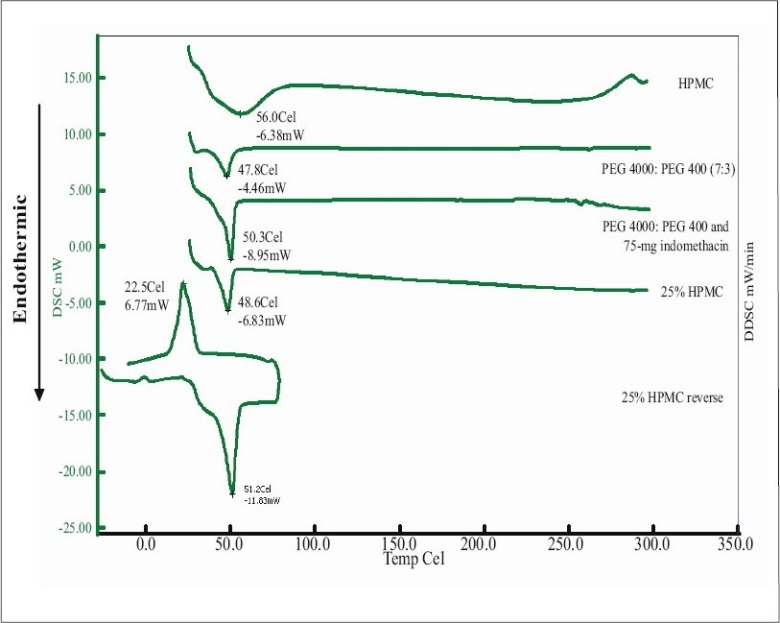
DSC thermograms of HPMC and indomethacin tablets. Differential scanning calorimetric thermograms of HPMC, indomethacin tablet containing 25% HPMC in 70:30 PEG 4000:PEG 400 system and reverse run DSC study for indomethacin tablet containing 25% HPMC in 70:30 PEG 4000:PEG 400 system.

Differential scanning calorimetry (DSC) was conducted to indicate the molecular dispersion of indomethacin into carrier matrix. Indomethacin and system containing 7:3 PEG4000:PEG400 exhibited the endothermic peak at 161.4° and 48° (fig. 4) whereas that of pure PEG4000 and pure PEG400 occurred at 58.9° and 58.2°, respectively (data not shown). DSC data for pure HPMC exhibited melting point at 56.2°. The DSC thermogram of system contained HPMC showed a melting point at 50.7°. The thermograms of the solid dispersion showed the characteristic peak of the carrier matrix (fig. 5), without drug peak indicating that the drug was completely dissolved in the molten carrier during DSC measurement. Analogous phenomena have also previously been reported[6,25,26]. Alteration of this drug in PEG-based SD system has been reported previously[27]. Thermograms of the PEG-based system showed the characteristic peak of the carrier matrix around 50°, but without the drug endothermic melting peak, indicating that the drug was changed into amorphous structure. The exothermic peak of tablets containing 75 mg indomethacin exhibited at 24.7° after reverse run to -30°. Because system containing 75 mg indomethacin contained a high amount of carrier, the carrier was recrystallized which the exothermic peak was occurred[28].

**Fig. 6:**
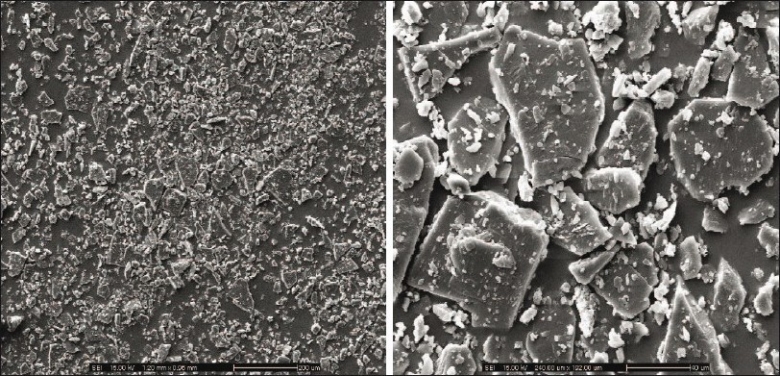
SEM micrographs of indomethacin powder. Scanning electron micrographs of indomethacin powder at magnification of 100X on the left and 500X on the right

**Fig. 7:**
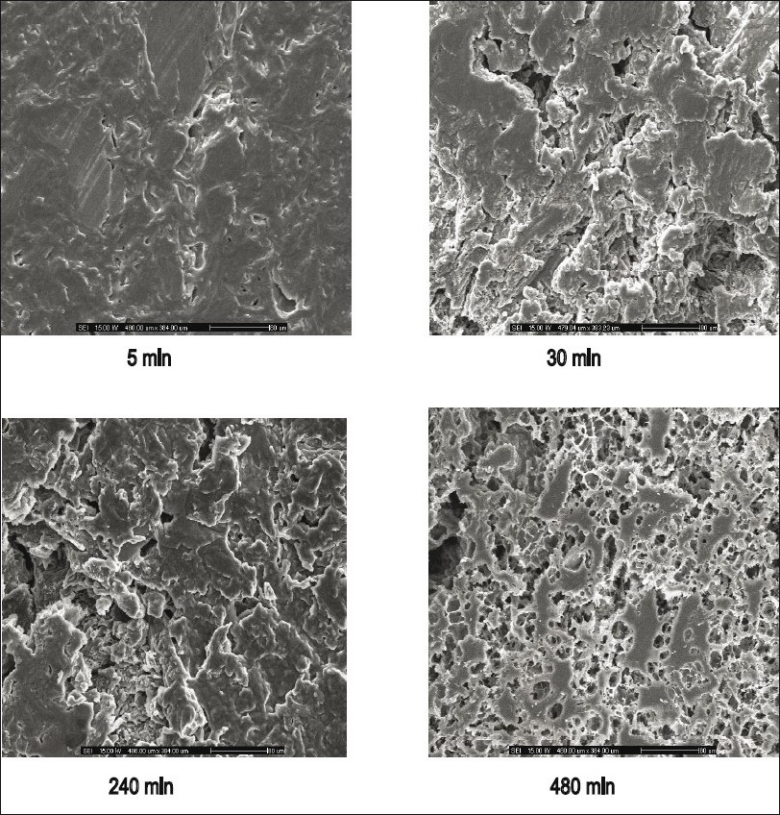
SEM micrographs of tablets of 75 mg indomethacin. Scanning electron microscope micrographs of tablets containing 75 mg indomethacin and 25% HPMC in 70:30 PEG 4000:PEG 400 system after dissolution test in phosphate buffer pH 6.2 at different time interval with magnification of 250X.

Micrographs of indomethacin powder are shown in fig. 6. Indomethacin was formed by plate crystals with smooth borders, but irregularly shaped and this morphology was retained also at small size. SEM images of tablet containing highest amount of HPMC exhibited the higher eroded surface with dissolution time and contained some pores after dissolution test at different time intervals (fig. 7).

Most likely, the morphological change was due to a leaching out of the water soluble polymers, PEG, HPMC, and the release of the indomethacin. This morphological characteristic change of other matrix has been previously claimed[29]. Porosity of the tablet containing 25% HPMC was increased with time. Therefore, dissolved drug molecules could diffuse through this pore so that the kinetic of drug release should be the diffusion-controlled release[30]. The dissolution enhancement of a poorly soluble model drug, rofecoxib, using solid dispersion approach has been described[6]. Solid state characterization revealed the partial loss of drug crystallinity which could bring about significant change to increase the drug dissolution.

To analyze the *in vitro* release data, the curve fitting of drug dissolution profiles to various kinetic models were carried out to describe the release kinetics. The coefficient of determination (r^2^) was used to indicate the degree of curve fitting. Goodness-of-fit was also evaluated using MSC. The zero order describes the systems where the drug release rate is independent of its concentration[31]. The first order describes the release from system where release rate is concentration dependent[32]. Higuchi described the release of drugs from insoluble matrix as a square root of time dependent process based on Fickian diffusion. Fickian diffusional release and a case-II relaxational release are the limits of this phenomenon. Fickian diffusional release occurs by the usual molecular diffusion of the drug due to a chemical potential gradient. Case-II relaxational release is the drug transport mechanism associated with stresses and state-transition in hydrophilic glassy polymers which swell in water or biological fluids. This term also includes polymer disentanglement and erosion[33].

The r^2^ from curve fitting to power law equation was in range 0.9881 to 0.9973 and msc was in range 4.01 to 4.71 (Table 2). From curve fitting, the drug release from tablets containing different amount of HPMC were fitted well with first order model since r^2^ and msc from curve fitting were higher than Higuchi's model and zero order curve fitting. ND means “not determined” for cumulative drug release which was less than 5% or more than 80% and that data points were not enough to be determined by curve fitting. Practically, all the kinetic models, other than the zero order, fitted well at early time periods. Therefore, modeling analysis was carried out by fitting the dissolution data until the time 80% of the drug released. However, in cases where the total drug release in the entire dissolution time span was below 80%, data until the last sampling time was selected as previously described[6].

**TABLE 2:** COMPARISON OF DEGREE OF GOODNESS-OF-FIT FROM CURVE FITTING

Formula	Power law		First order		Higuchi's		Zero order	
				
	r^2^	msc	r^2^	msc	r^2^	msc	r^2^	msc
Capsule	0.9913	4.16	0.9955	2.98	0.9811	3.51	0.9915	4.23
5% HPMC	ND	ND	ND	ND	ND	ND	ND	ND
10% HPMC	ND	ND	ND	ND	ND	ND	ND	ND
15% HPMC	0.9973	4.71	0.9998	7.85	0.9861	3.48	0.9675	2.63
20% HPMC	0.9915	3.23	0.8313	1.49	0.7813	1.23	0.6632	0.80
25% HPMC	0.9881	4.01	0.9774	3.51	0.9880	4.14	0.9662	3.10

Comparison of degree of goodness-of-fit from curve fitting of drug dissolution from tablet containing different amounts of HPMC in 70:30 PEG 4000:PEG 400 system in phosphate buffer pH 6.2 to different release models. ND: not determined

The exponent *(n)* values for almost formula are shown in Table 3. The magnitude of the exponent *n* could indicate the release mechanism such as Fickian diffusion, case II transport, or anomalous transport. In the present study (cylindrical shape) the limits considered were *n* = 0.45 (indicates a classical Fickian diffusion-controlled drug release) and *n* = 0.89 (indicates a case II relaxational release transport: polymer relaxation controls drug delivery). Values of *n* between 0.45 and 0.89 can be regarded as indicators of both phenomena (transport corresponding to coupled drug diffusion in the hydrated matrix and polymer relaxation), commonly called anomalous non-Fickian transport[34]. The k value can be regarded as power law release constant with bigger values leading to faster drug release. Mathematically, increase in *n* values bigger than 1.0 would allow for a more desirable incremental release. To achieve delayed release pattern that was therapeutically useful, *n* values were expected to be bigger and *k* values to be smaller with a prerequisite of acceptable lag time[35].

**TABLE 3:** ESTIMATED PARAMETERS FROM CURVE FITTING OF DRUG DISSOLUTION FROM TABLETS

Formula	k±SD*10^-1^	tl±SD (min)	n±SD
Capsule	0.1232±0.0208	3.12±1.03	0.31±0.05
5% HPMC	ND	ND	ND
10% HPMC	ND	ND	ND
15% HPMC	0.0811±0.0175	2.43±1.16	0.56±0.05
20% HPMC	0.0270±0.0290	6.12±1.31	0.47±0.03
25% HPMC	0.0221±0.0061	-18.64±12.80	0.48±0.05

Estimated parameters from curve fitting of drug dissolution from tablet containing different amount of HPMC in 70:30 PEG 4000:PEG 400 system in phosphate buffer pH 6.2 to power law expression. ND: not determined

From curve fitting to power law equation, the *n* values of tablet containing different amount of HPMC indicated that the release mechanism of these tablets were close to anomalous transport. Therefore, mechanism and kinetics of drug release are dependent on the solubility of the active moiety and the swelling and erosion properties of the polymer, with water soluble drugs being released predominantly by diffusion with a limited contribution from matrix erosion and anomalous diffusion resulting from the relaxation of the macromolecular polymer chains[36]. Therefore, both the enhancement of drug dissolution and the prolongation of the drug release could be achieved for poorly water-insoluble drug such as, indomethacin, by using PEG-HMC matrix system prepared with fusion and mold technique. Least square curve fitting indicated that the drug liberated from the developed matrix system conformed to an anomalous transport.
